# Author Correction: Identification of genetic factors influencing metabolic dysregulation and retinal support for MacTel, a retinal disorder

**DOI:** 10.1038/s42003-021-01972-y

**Published:** 2021-04-09

**Authors:** Roberto Bonelli, Victoria E. Jackson, Aravind Prasad, Jacob E. Munro, Samaneh Farashi, Tjebo F. C. Heeren, Nikolas Pontikos, Lea Scheppke, Martin Friedlander, MacTel Consortium, Catherine A. Egan, Rando Allikmets, Brendan R. E. Ansell, Melanie Bahlo

**Affiliations:** 1 Population Health and Immunity Division, Walter and Eliza Hall Institute of Medical Research, Parkville, VIC Australia; 2 Department of Medical Biology, University of Melbourne, Parkville, VIC Australia; 3 Moorfields Eye Hospital NHS Foundation Trust, London, UK; 4 University College London Institute of Ophthalmology, London, UK; 5 The Lowy Medical Research Institute, La Jolla, CA USA; 6 Department of Molecular Medicine, The Scripps Research Institute, La Jolla, CA USA; 7 Department of Ophthalmology, Columbia University, New York, NY USA; 8 Department of Pathology and Cell Biology, Columbia University, New York, NY USA

**Keywords:** Gene expression, Genome-wide association studies

Correction to: *Communications Biology* 10.1038/s42003-021-01788-w, published online 2 March 2021.

In the original version of the Article, the following texts shown in bold was inadvertently included in the second paragraph of the “Methods” subsection “UK Biobank analysis”: “Grading was performed on 200 subjects reporting no eye conditions **as well xxx reporting ‘other serious eye conditions’. The last were selected since they presented on average higher MaCtel PRS scores compared to all other reported retinal disorders**”.

In the original version of the Article, the clinical description of MacTel patients in the Introduction was incorrect. The final sentence of the first paragraph in the Introduction incorrectly read, “MacTel patients experience blurred or lack of peripheral vision, which progresses to complete loss of central vision.” The correct text is, “MacTel patients may initially experience blurred or fragmented central vision, which may slowly progress to more significant loss of central and/or paracentral vision.” The errors have been corrected in the HTML and PDF versions of the Article.

