# Supplementary material for: Identification of genetic factors influencing metabolic dysregulation and retinal support for MacTel, a retinal disorder
Source: Commun Biol. 2021 Mar 2;4:274. doi: 10.1038/s42003-021-01788-w (PMC7925591; doi:10.1038/s42003-021-01788-w)
Supplement: Supplementary file 3 — Description of Additional Supplementary Files [file 42003_2021_1788_MOESM3_ESM.pdf]

Description of additional supplementary files

File name: Supplementary Data 1 – 18
Description: Excel file containing the supplementary datasets

Supplementary Data 1: Summary of SNP and Sample-based quality control:
A. Tranche 1 - MacTel consortium samples genotyped on Omni5-exome (pre-imputation);
B. Tranche 2 - AREDs controls genotyped on Omni-2.5 (pre-imputation);
C. Tranche 3 - MacTel Consortium and Twinning samples genotyped on the Global Screening Array (pre-imputation);
D. All tranches merged (post-imputation).

Supplementary Data 2: Sample composition and genotyping platform for cases and controls included in the full cohort, and European-only sub-set.

Supplementary Data 3: 95% credible sets of causal SNPs at each GW-significant locus, as determined by Bayesian fine-mapping.[1]

Supplementary Data 4: Haplotype frequencies for the two *PHGDH* SNPs (rs146953046 and rs532303), in cases and controls. The alleles associated with increased MacTel risk are the G allele for rs146953046 and the A allele for rs532303.

Supplementary Data 5: Sex Interaction Analysis. Association results for the 11 genome-wide significant SNPs, in males and females separately. For each SNP, sex-interaction was formally tested using Welch's t-test.

Supplementary Data 6: MAGMA results tables as performed by FUMA. Only genes with FDR<0.05 are shown. NSNPs is the number of SNPs used by MAGMA to calculate principal components for each gene. The number of PCs used for each gene is presented in NPARAM.

Supplementary Data 7: List of parameters used in FUMA. eQTL tissues can be found in parameter "eqtlMaptss".

Supplementary Data 8: Summary of eQTL results table. This table presents the average effect of all MacTel risk SNPs affecting gene expression for each gene in each tissue. nSNP is the significant eSNPs for each gene in each tissue. nTissue is the count of tissue presenting significant eSNPs for each gene. Global mean effect is the average among tissue specific effects. Direction indicated whether the gene should be positively or negatively regulated by the SNPs increasing MacTel risk.

Supplementary Data 9: Genetic colocalization results for MacTel genome-wide significant loci compared to retinal eQTL loci and GW-significant loci for MacTel-related traits. Candidate gene: gene bounding, or closest to MacTel GWAS locus; N. SNPs: number of SNPs represented in both GWAS / eQTL result sets used for colocalization analysis; PP shared causal variants: posterior probability that the Mactel GWAS and Retina eQTL/related trait GWAS signals derive from a shared variant; PP distinct causal variants: Posterior probability that the Mactel GWAS and Retina eQTL/related trait GWAS signals derive from distinct variants. P-values in bold meet the gene-specific threshold for a significant eQTL as determined by Ratnapriya et al.[2]. Posterior probabilities for shared causal variants >0.75 (bold text) are considered strong evidence of genetic colocalization.

Supplementary Data 10: List of significant and suggestive-significant genes for each tissue from the TWAS.

Supplementary Data 11: List of prioritised genes by any analysis. GW_sig_snp is 1 if the gene was located underneath a locus reaching GW significance. Sugg_GW_sig_snp is 1 if the gene was located underneath a locus reaching GW significance or suggestive significance. eQTL is 1 if the gene was significantly affected in any tissue by any SNPs in LD (r2>0.4) with significant or suggestive significant loci. MAGMA is 1 if the gene was significant in the MAGMA analysis. TWAS is 1 if the gene was significant in the TWAS analysis. N_evidence is the sum of all evidence.

Supplementary Data 12: Retina vs RPE tissue expression comparison results based on Whitmore et al 2014 *Exp Eye Res*[3]. Additionally, comparison between nasal (NAS), temporal (TMP) and macular (MAC) regions of the retina/RPE is presented for all genes.

Supplementary Data 13: Single cell expression comparison results based on three studies detailed in Online Methods. The cell type exhibiting maximal expression for each gene is presented in cell_max_expression. Average increase of expression between that cell type and all other cell types is represented in the Effect column.

Supplementary Data 14: LD-score regression results from LDhub database and an additional 13 ocular phenotypes not included in LDhub. Traits with a significant genetic correlation with MacTel are those with p-values below 0.05. Reference study details are provided in online methods. LDhub databases and studies contain subjects of predominantly European ancestry. SNPs from the major histocompatibility complex region (chr6 26M~34M) were excluded from this analysis. SE: standard error; Z: z score statistics for correlation significance.

Supplementary Data 15: Mendelian Randomization results. Each tested metabolite PRS is presented in columns Metabolite. Effect of genetically predicted metabolic abundance on disease risk is presented in column effect and OR.

Supplementary Data 16: MacTel Consortium members and affiliations

Supplementary Data 17: Copy of Table 1 from the main manuscript

Supplementary Data 18: Copy of Table 2 from the main manuscript

References

1. Hutchinson, A., Watson, H. & Wallace, C. Improving the coverage of credible sets in Bayesian genetic fine-mapping. *PLoS Comput. Biol.* **16**, e1007829 (2020).

2. Ratnapriya, R. *et al.* Retinal transcriptome and eQTL analyses identify genes associated with age-related macular degeneration. *Nat. Genet.* **51**, 606–610 (2019).

3. Whitmore, S. S. et al. Transcriptomic analysis across nasal, temporal, and macular regions of human neural retina and RPE/choroid by RNA-Seq. *Exp. Eye Res.* **129**, 93–106 (2014).
