# Supplementary material for: Identification of genetic factors influencing metabolic dysregulation and retinal support for MacTel, a retinal disorder
Source: Commun Biol. 2021 Mar 2;4:274. doi: 10.1038/s42003-021-01788-w (PMC7925591; doi:10.1038/s42003-021-01788-w)
Supplement: Supplementary file 5 — Reporting Summary [file 42003_2021_1788_MOESM5_ESM.pdf]

nature research

Corresponding author(s): Prof. Melanie Bahlo
bahlo@wehi.edu.au

Last updated by author(s): 14 December 2020

Reporting Summary

Nature Research wishes to improve the reproducibility of the work that we publish. This form provides structure for consistency and transparency in reporting. For further information on Nature Research policies, see our Editorial Policies and the Editorial Policy Checklist.

Statistics

For all statistical analyses, confirm that the following items are present in the figure legend, table legend, main text, or Methods section.

n/a	Confirmed	
☐	☒	The exact sample size (*n*) for each experimental group/condition, given as a discrete number and unit of measurement
☐	☒	A statement on whether measurements were taken from distinct samples or whether the same sample was measured repeatedly
☐	☒	The statistical test(s) used AND whether they are one- or two-sided<br>*Only common tests should be described solely by name; describe more complex techniques in the Methods section.*
☐	☒	A description of all covariates tested
☐	☒	A description of any assumptions or corrections, such as tests of normality and adjustment for multiple comparisons
☐	☒	A full description of the statistical parameters including central tendency (e.g. means) or other basic estimates (e.g. regression coefficient) AND variation (e.g. standard deviation) or associated estimates of uncertainty (e.g. confidence intervals)
☐	☒	For null hypothesis testing, the test statistic (e.g. *F*, *t*, *r*) with confidence intervals, effect sizes, degrees of freedom and *P* value noted<br>*Give P values as exact values whenever suitable.*
☒	☐	For Bayesian analysis, information on the choice of priors and Markov chain Monte Carlo settings
☒	☐	For hierarchical and complex designs, identification of the appropriate level for tests and full reporting of outcomes
☐	☒	Estimates of effect sizes (e.g. Cohen's *d*, Pearson's *r*), indicating how they were calculated

Our web collection on statistics for biologists contains articles on many of the points above.

Software and code

Policy information about availability of computer code

Data collection	No software was used for the data collection of this study.
Data analysis	R (v3.6.1), PLINK (v1.90b3w), Michigan Imputation Server (v1.0.4), BCFtools (v1.7), GENESIS (R package v2.14.3), SNPRELATE (R package v1.18.1), SAIGE v0.36.2, GCTA (v1.93.2), Locuszoom (v1.4), FUMA (v1.3.5e), MAGMA (v1.07), LD hub (v1.9.3), LDSC(v1.0.1 and v1.1), TWAS-FUSION (vNA), PRSice-2 (v2.3.1.e), subread (v1.6.3), ROCR (R package v1.07), pROC (R package v1.16.1), coloc (R package v3.2.1), limma (R package v3.4). In house R-scripts are available upon request.

For manuscripts utilizing custom algorithms or software that are central to the research but not yet described in published literature, software must be made available to editors and reviewers. We strongly encourage code deposition in a community repository (e.g. GitHub). See the Nature Research guidelines for submitting code & software for further information.

Data

Policy information about availability of data

All manuscripts must include a data availability statement. This statement should provide the following information, where applicable:
- Accession codes, unique identifiers, or web links for publicly available datasets
- A list of figures that have associated raw data
- A description of any restrictions on data availability

GWAS summary statistics are available at doi:10.17605/OSF.IO/GEK7B. Control genotyping data were sourced from dbGaP for the National Eye Institute (NEI) Age-Related Eye Disease Study (AREDS) (dbGaP accession number phs000001.v3.p1). Further control genotyping data were sourced from the QIMR Genetics of Twins study (Professor Nick Martin, available on request). Raw RNA sequencing for human retina accessed via GEO GSE115828, and genotype data for the same subjects were generously provided by Prof Anand Swaroop and Dr Rinki Ratnapriya of the US NEI, subject to Material Transfer Agreement. MacTel patient genotyping data and a subset of the controls are available from the European Genome and Phenome archive (EGAS00001002249).

Field-specific reporting

Please select the one below that is the best fit for your research. If you are not sure, read the appropriate sections before making your selection.

☒ Life sciences ☐ Behavioural & social sciences ☐ Ecological, evolutionary & environmental sciences

For a reference copy of the document with all sections, see nature.com/documents/nr-reporting-summary-flat.pdf

Life sciences study design

All studies must disclose on these points even when the disclosure is negative.

Sample size	1067 MacTel patients and 3799 controls were included in the GWAS discovery analysis. 200 UKBiobank patients (100 randomly selected from the top MacTel PRS decile and 100 in the bottom MacTel PRS decile) who self-reported absence of any eye conditions.
Data exclusions	Samples were excluded if they failed quality control, as described in the methods. Some individuals from the MacTel consortium were excluded, as MacTel disease status could not be confirmed. UK Biobank selected participants with OCT scans presenting an image quality too low to be graded were excluded from the study.
Replication	No replication was performed in this study; MacTel is a rare disease, and as such no replication samples were available at this time.
Randomization	We randomized cases and controls to make sure that they were distributed evenly across library preparation dates
Blinding	Blinding was not performed for the GWAS or post GWAS analysis. Ophthalmologists were blinded on the PRS status when grading retinal damage on the UK Biobank participants.

Reporting for specific materials, systems and methods

We require information from authors about some types of materials, experimental systems and methods used in many studies. Here, indicate whether each material, system or method listed is relevant to your study. If you are not sure if a list item applies to your research, read the appropriate section before selecting a response.

Materials & experimental systems

n/a	Involved in the study	
☒	☐	Antibodies
☒	☐	Eukaryotic cell lines
☒	☐	Palaeontology and archaeology
☒	☐	Animals and other organisms
☐	☒	Human research participants
☒	☐	Clinical data
☒	☐	Dual use research of concern

Methods

n/a	Involved in the study	
☒	☐	ChIP-seq
☒	☐	Flow cytometry
☒	☐	MRI-based neuroimaging

Human research participants

Policy information about studies involving human research participants

Population characteristics	The discovery GWAS sample was predominantly of European ancestry (3,745 controls, 98%; 931 cases, 87%), with a higher proportion of females (78% in controls; 60% in cases). A breakdown by tranche is given in table S2.
Recruitment	Participants from the MacTel Consortium (Mactel cases, and unaffected family members and spouses as controls) were recruited from 23 clinical centres, from seven countries. A complete list of participating institutes are provided in the Supplementary Note. Criteria for MacTel diagnosis are described in the Online Methods. The QIMR Twinning Genetics Study samples were recruited via the Australian and Dutch Twin Registries and public appeals. AREDs controls were accessed via dbGaP (phs000429.v1.p1).
Ethics oversight	For the MacTel Consortium recruitment, a full list of approving ethics committees are provided in the Supplementary Note. The QIMR Twinning Genetics study was approved by the Human Research Ethics Committee of the QIMR Berghofer Medical Research Institute.

Note that full information on the approval of the study protocol must also be provided in the manuscript.
